# Supplementary material for: The Expression of Cannabinoid and Cannabinoid-Related Receptors on the Gustatory Cells of the Piglet Tongue
Source: Molecules. 2024 Sep 28;29(19):4613. doi: 10.3390/molecules29194613 (PMC11478043; doi:10.3390/molecules29194613)
Supplement: Supplementary file 1 [file molecules-29-04613-s001.zip › molecules-3182257-supplementary.pdf]

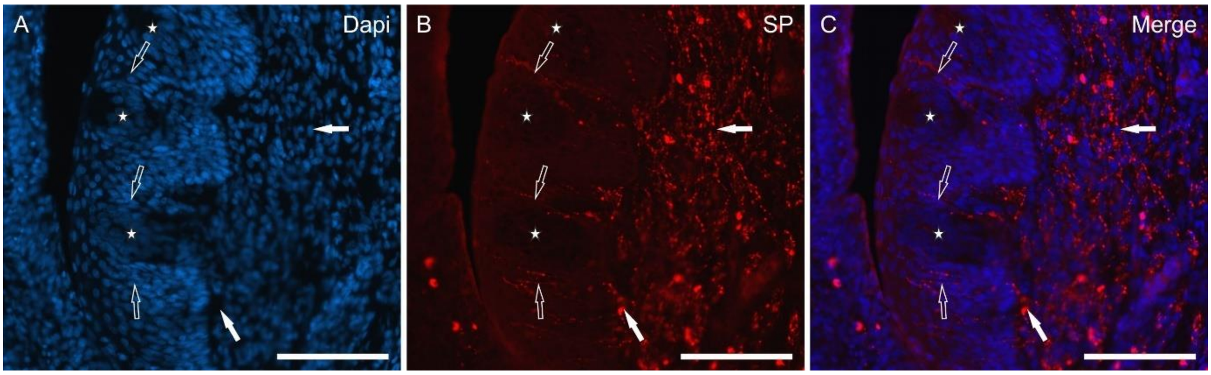


**Supplementary figure S1.** Photomicrographs showing a cryosection of the piglet papilla vallata in which the anti- substance P (SP) antibody (B) was applied. The empty arrows indicate thin nerve varicosities immunoreactive for substance P (SP) (B), adjacent to some cells belonging to three contiguous taste buds (stars). The white arrows indicate nerve fibers and varicosities immunoreactive for SP concentrated in the connective tissue underlying the papillae. C: merge image. Scale bar: 50 μm.

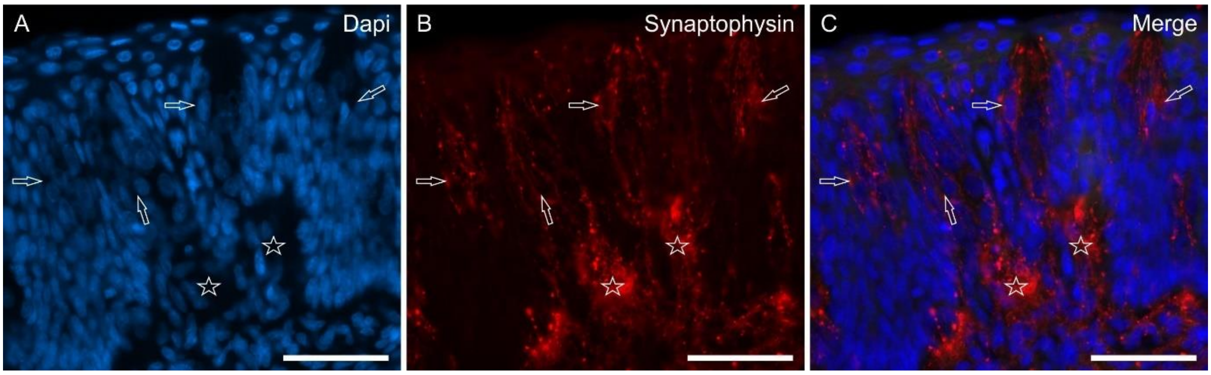


**Supplementary figure S2.** Photomicrographs showing a cryosection of the piglet papilla vallata in which the anti-synaptophysin antibody was applied. The arrows indicate thin nerve varicosities, adjacent to some cells belonging to four contiguous taste buds, which were synaptophysin immunoreactive. The stars indicate particularly dense synaptophysin-positive nerve fibers and varicosities at the base of contiguous taste buds. C: merge image. Scale bar: 50 μm.

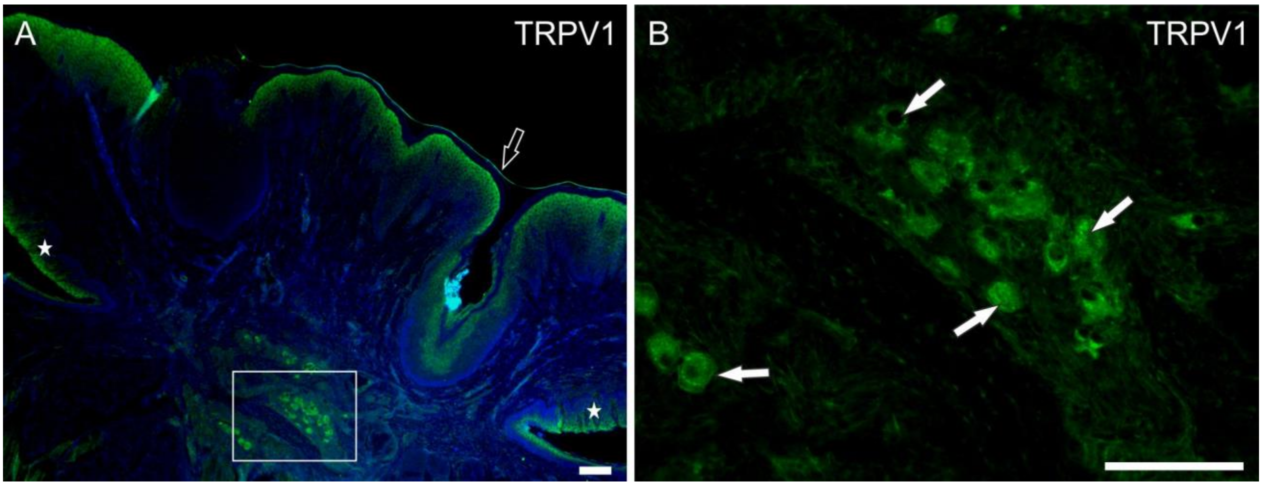


**Supplementary Figure S3**. Photomicrographs showing a large piglet papilla vallata in which the anti-TRPV1 antibody was applied. A) The white frame (its magnification is Figure B) delimits the neuronal ganglia just located beneath the base of the papilla the neurons of which (white arrows, B) expressed TRPV1 immunoreactivity. The stars indicate some taste buds. The open arrow indicates the bunching (retraction) of the mounting media close to the tissue surface causing a faint autofluorescence line. Scale bar: 50 μm.

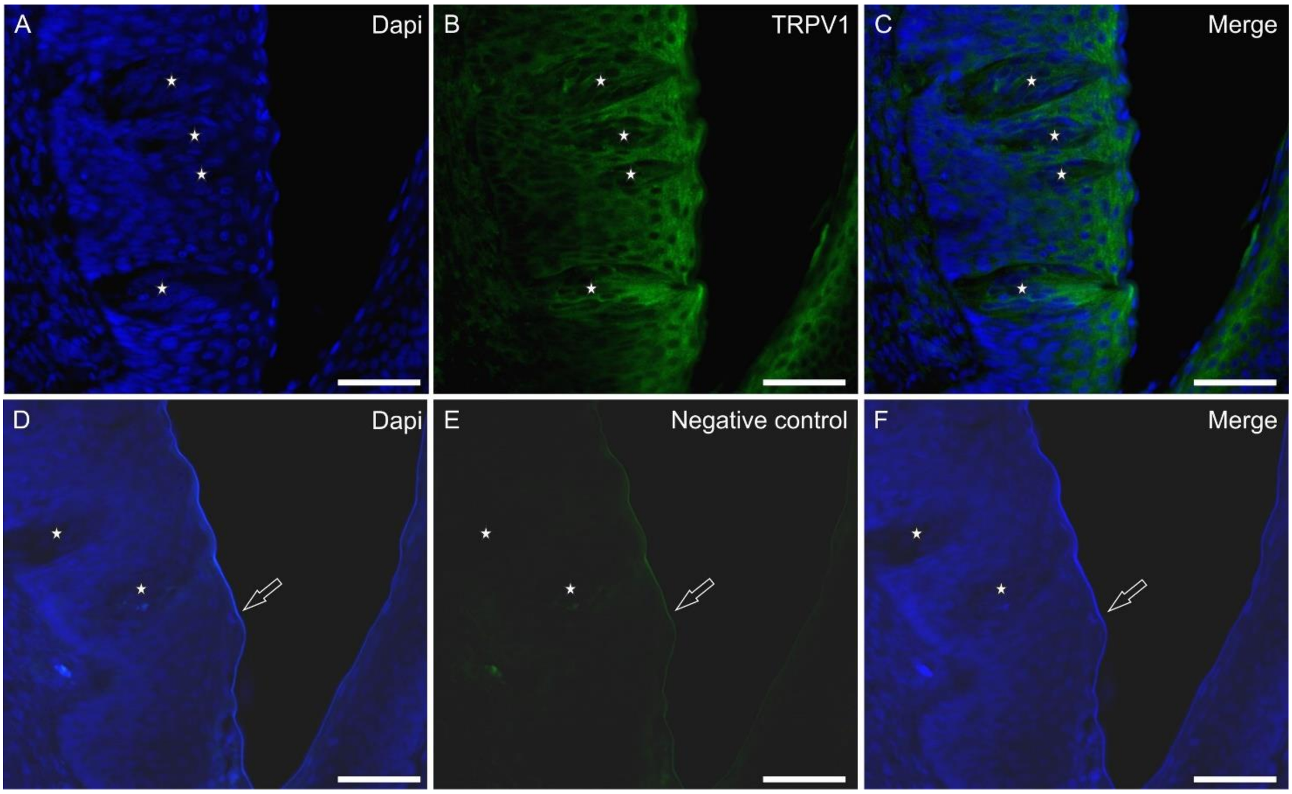


**Supplementary Figure S4**. Photomicrographs showing two adjacent cryosections of the piglet papilla vallata. A-C) On this section, the anti-TRPV1 antibody was applied; D-F) Only the secondary antibody was applied on this section (negative control), and no TRPV1 immunolabeling is visible. The stars indicate taste buds. The arrows indicate the bunching (retraction) of the mounting media onto the tissue surface causing a faint autofluorescence line. C and F: merge images. Scale bar: 50 μm.
